# Taxonomy bias in metagenome-assembled genome recovery

**DOI:** 10.1099/mgen.0.001736

**Published:** 2026-06-08

**Authors:** Daniel J. Nebauer, Tiffanie Nelson, Caitlin Romanis, Brett A. Neilan, Verlaine J. Timms

**Affiliations:** 1 School of Environmental and Life Sciences, The University of Newcastle, Callaghan, New South Wales, Australia; 2 Australian Research Council Centre of Excellence in Synthetic Biology, Macquarie Park, NSW, 2109, Australia; 3 Faculty of Medicine, Dentistry and Health Sciences, The University of Melbourne, Melbourne, Victoria, Australia

**Keywords:** GC bias, metagenome-assembled genomes (MAGs), microbial diversity, shotgun metagenomics, taxonomic bias

## Abstract

The recovery of metagenome-assembled genomes (MAGs) from shotgun metagenomic sequencing is rapidly expanding the availability of representative genomes. However, this practice may skew the representation of specific taxa in real-world datasets. This bias is attributed primarily to the known inefficiencies of sequence-by-synthesis platforms in amplifying GC-rich and AT-rich sequence fragments. Here, we recover 216 medium- and high-quality MAGs from an Australian wetland site. Notably, no MAGs were recovered for some dominant cyanobacterial and proteobacterial species known to be present. A new protocol involving read-based classification and alignment to the MAG dataset demonstrated the highly efficient recovery of low-GC organisms in the *Actinobacteria* and *Bacteroidota* phyla. Additionally, the recovery of lost taxonomic information was demonstrated through unmatched sample mapping. The findings suggest a bias towards the recovery of smaller, low-GC organisms in MAG recovery, potentially skewing the global representation of microbial diversity. Our pipeline is made publicly available as a tool to help researchers estimate taxonomic losses following MAG recovery efforts.

**Impact Statement**

Metagenome-assembled genomes (MAGs) have become central to genome-resolved microbial ecology, yet the potential biases introduced by assembly and binning remain underexplored in real-world datasets. This study demonstrates that MAG recovery does not necessarily reflect organismal abundance, revealing systematic taxonomic distortions linked to GC content and genome size. Using shotgun metagenomes from an Australian wetland, we show preferential recovery of smaller, low-GC genomes and the loss of dominant taxa, including ecologically significant *Cyanobacteria*. We further introduce a read-based mapping strategy that partially restores lost taxonomic information, providing a practical framework for assessing MAG recovery efficiency. These findings highlight an important limitation of current MAG-centric analyses and offer methodological guidance to improve the interpretation of genome-resolved metagenomic studies.

## Data Availability

All raw shotgun metagenomic sequencing data generated in this study are publicly available through the NCBI Sequence Read Archive (SRA) under BioProject accession PRJNA1230272.

The ten individual SRA accessions are SRR32538009–SRR32538018.

Scripts for data analysis and visualization are publicly available in the mag-attrition-tool repository: https://github.com/DanielNebauer94/mag-attrition-tool.

## Introduction

Shotgun metagenomics is an established but evolving field enabling the genomic profiling of microbial communities. It increases taxonomic depth and resolution over 16S rRNA amplicon sequencing approaches [1], while also providing insights into community functions [2]. Of increasing importance in microbial ecology is the recovery of metagenome-assembled genomes (MAGs), which are reconstructions of complete or near-complete genomes from shotgun metagenomic data [3]. MAG recovery has revolutionized the discovery of a vast number of novel microbes which are not amenable to cultivation and has seen a substantial expansion of the tree of life [4,5]. Further, MAG recovery circumvents time-consuming isolation and sequencing of single organisms, enabling genomic profiling of microbes often at the genus or species level, and in some instances may discriminate between strains or ecotypes [6].

MAG recovery requires four essential steps: (1) DNA from a microbial community is sequenced into reads, (2) reads are assembled into longer contiguous sequences called contigs, (3) contigs are grouped together based on sequence similarity, a practice called binning, and (4) bins are assessed for completion and contamination and taxonomy is inferred through phylogenetic placement into the tree of life [7]. Bioinformatic pipelines to bin contigs and ease MAG recovery are rapidly evolving [8]; however, most current algorithms bin contigs based on a combination of tetranucleotide frequencies and contig coverage values [9].

As MAGs rapidly expand the global catalogue of microbial genomes [4], it is becoming increasingly important to consider how this technology may be skewing the representation of specific taxa. The GC content of bacterial genomes can vary widely and significantly affect sequencing and assembly efforts [10, 11]. High-throughput sequencing platforms which employ sequence by synthesis technologies [12] are known to inefficiently amplify both GC and AT-rich sequence fragments [13,14]. Reduced read coverage of GC-extreme regions results in fragmented assemblies, hindering the recovery of high-quality genomes from organisms with extreme GC content [15]. In the context of MAG recovery, fragmented assemblies reduce the genomic template available for robust assessment of tetranucleotide frequencies during binning [16], theoretically disfavouring recovery of genomes with extreme GC content at the sequencing and assembly stages. While GC bias is well-documented in the recovery of GC-extreme microbes and mock community datasets [15,17], little attention has been given to its potential consequences in real-world metagenomic datasets. Further, very few bioinformatic tools exist to examine this problem in a straightforward manner.

Here, we employ a widely adopted pipeline to recover MAGs from shotgun metagenomic sequencing data generated in a real-world, year-long study investigating a wetland site in Australia. The site experienced high season-to-season taxonomic variability, with one sample dominated by *Planktothrix*, an AT-rich genus of *Cyanobacteria* [18]. We identify taxonomic information lost during MAG recovery and investigate the role of GC content. We also demonstrate the use of read alignment to unmatched samples as a viable way to recover taxonomic information lost during MAG recovery. We make this pipeline publicly available as a practical tool for researchers to quantify taxonomic attrition following MAG recovery efforts, deployable on modest computational infrastructure without the need for high-performance computing resources.

## Methods

### Sample collection

Water samples were collected according to the sample key in Table 1. Sampling sites were termed ‘Inlet’ due to the proximity to an inlet pipe and ‘Colony’ due to the proximity to a flying fox colony. Water samples were collected with a sterilized polypropylene bottle. The bottle was washed three times with water close to the sample site prior to sample collection and care was taken not to disturb the water column. Biomass was filtered onto 1.2 µm glass fibre filters within 5 h of collection. Filters containing biomass were stored at −30 °C in 1.5 ml Eppendorf tubes until required.

**Table 1.** Sample key. A total of 10 samples underwent shotgun metagenomic sequencing. Samples encompassed two sites (Inlet and Colony) and five dates, which spanned 11 months

Sample name	Site	Date
RW01	Inlet	16/06/2020
RW02	Inlet	08/09/2020
RW03	Inlet	29/12/2020
RW04	Inlet	23/02/2021
RW05	Inlet	08/04/2021
RW06	Colony	16/06/2020
RW07	Colony	08/09/2020
RW08	Colony	29/12/2020
RW09	Colony	23/02/2021
RW10	Colony	08/04/2021

### Genomic DNA extraction, sequencing and quality control

Genomic DNA extraction from filters was performed using the FastDNA Spin Kit for Soil (MP Bio, Solon, Ohio), following the manufacturer’s instructions. DNA was quantified using the high-sensitivity Qubit dsDNA assay (Invitrogen). Paired-end sequencing and demultiplexing was performed at the Ramaciotti Genomic Research Centre (UNSW) using a NovaSeq (Illumina) with a NovaSeq 6000 SP reagent kit (300 cycles) at a read length of 150 bp. Quality trimming and the removal of adapters and unpaired reads were performed using Trimmomatic v0.32 [19], with the following arguments: -phred33 -leading:3 -trailing:3 -slidingwindow:4 : 15 -minlen:36. Read quality in each sample was assessed with FastQC (v0.12.1) and visualized with MultiQC (v1.16), with phred scores >20 used as the metric for quality passing, which allows 1% base call error [20].

### Assembly, binning, MAG recovery and relative abundance

All analyses were performed on a workstation running Windows 10 with the Windows Subsystem for Linux (WSL), equipped with an Intel Core i5-12400F processor, 128 GB DDR4 RAM, and a 3 TB external soild state drive.

The appropriate algorithm for assembly and binning depends on the dataset [21,22], so two methods for assembly and three methods for binning were compared. High-quality reads were assembled into contigs/scaffolds with metaSPAdes v3.15.2 and MEGAHIT v1.1.4. A co-assembly using either the MEGAHIT or metaSPAdes assemblers was not possible using the available computational infrastructure [23], so each dataset was assembled individually. MetaQUAST v5.2.0 [24] was run in reference-free mode to assess assembly quality.

Contigs underwent binning via the metabat2 v2.15 [25], maxbin2 v2.2.7 [26] and metaWRAP v1.3 pipelines, with minimum contig length set at 1,000 bp. Bin completion and contamination metrics were computed using checkM v1.2.2 and a set of 43 phylogenetically informative marker genes [27]. This study followed minimum information about a MAG standards to define genome quality [28]; ≥50% completion and ≤10% contamination were defined as medium quality, and ≥90% completion and ≤5% contamination were defined as high quality. Low-quality MAGs (≤50% completion and/or ≥10% contamination) were removed. Bins were classified with GTDB-Tk v2.3.2 through placement into a reference tree against genomes from the GTDB database (release 207) [7]. At this point, they were defined as MAGs.

MAG relative abundance was computed using coverM v0.6.1 (https://github.com/wwood/CoverM). Contig edges were trimmed by 75 bp to avoid contig edge coverage bias [29], and MAGs with less than 10% total coverage were not considered. Relative abundance for each MAG was calculated as the number of reads aligned to a MAG divided by the total number of bacterial reads in each metagenomic dataset.

### Phylogenetic tree construction

A phylogenetic tree was constructed using GToTree v1.8.1 [30] and the gene set (HMM file) for bacteria (74 targets). Coding sequences were retrieved using Prodigal v2.6.3 [31], and HMMER3 v3.2.1 [32] was used to identify target genes present in the HMM file. Gene alignments were then performed by muscle v5.1 [33], and alignments were trimmed using Trimal v1.4 [34]. The concatenated aligned genes were then used to construct an approximate maximum likelihood tree using FastTree 2 v2.1.11 [35]. The tree was visualized and decorated using iTOL v6.9 [36].

### Taxa recovery estimations

High-quality reads were taxonomically classified with Kraken2 v2.1.1 [37], using a 64 GB kraken-compatible version of the GTDB database (created 23 July 2019) [38]; as such, nomenclature used throughout this manuscript follows GTDB conventions. Read classification results from the kraken2 kreport file were computed and visualized in R v4.2.0 with the Pavian package v1.2.1 [39].

To investigate read recovery into MAGs, bacterial reads were first extracted from fastq files with classification information obtained from kraken2 files. Extracted reads were then aligned to MAG datasets using BWA-MEM v0.7.18 [40] with default parameters. Samtools v1.2.0 [41] was used to convert resultant sequence alignment map (SAM) files to binary (BAM) format and to isolate aligned and unaligned reads into separate fastq files. For each genus in each sample, an in-house python script was used to determine the proportion of aligned vs unaligned reads and the average read GC content.

Barplots, density plots, bubble plots and heatmaps were generated in R v4.2.0 using the ggplot2 v3.5.1 [42] and pheatmap v1.0.12 [43] packages. Bubble plots were generated using a custom python script publicly available at https://github.com/DanielNebauer94/mag-attrition-tool.

## Results

### Read-based community composition

**Fig. 1.**
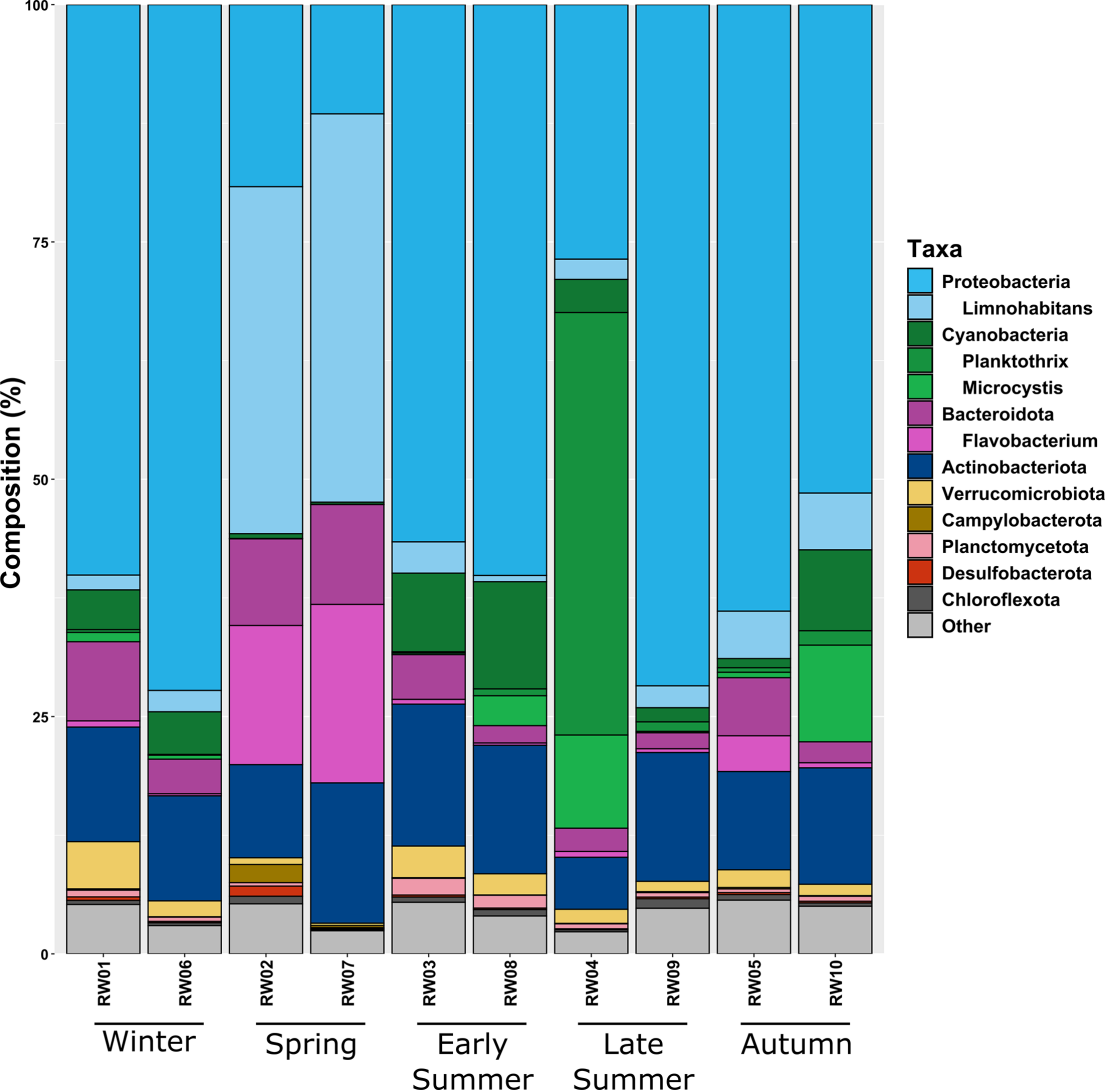
Bacterial community abundance. Bacterial abundance was determined according to metagenomic read-based taxonomic classification. Only reads classified as bacterial were considered, and thus the bar plot represents the relative abundance of each taxon as a proportion of classified bacterial reads only. Samples are clustered according to season. Phyla with >1% relative composition in at least one sample are presented. Also presented are genera with high abundance (>10%) in at least one sample. These genera are indented under their respective phyla in the taxa key.

We analysed ten metagenomic samples (RW01–RW10) collected from the Inlet and Colony sites across five seasonal time points. Reads were taxonomically classified using Kraken2 against a GTDB-compatible reference database (see methods). An average number of 42.9 million reads were obtained from all samples, ranging from 32.7 to 48.5 million. Of these, an average of 22.17% were classified as bacteria and archaea, with the highest percentage of read classification achieved during late summer at the Inlet site (RW09; 38.5%) and the lowest during autumn at the Colony site (RW10; 6.77%). Additional information about read quality and classification is available in the supplementary (Data S1 and S2, available in the online Supplementary Material). Read classification yielded 151 phyla, including 16 archaea. As this study focused on the bacterial community, non-bacterial reads were excluded from downstream analysis. Of classified bacterial reads, samples at both sites predominantly consisted of *Proteobacteria* (59%), *Cyanobacteria* (11%), *Actinobacteria* (11%) and *Bacteroidota* (9%) (Fig. 1). The relative abundance of *Cyanobacteria*, *Bacteroidota* and *Proteobacteria* was highly variable across samples, as were major genera such as *Planktothrix*, *Limnohabitans*, *Flavobacterium* and *Microcystis*.

The percentage of reads classified as *Cyanobacteria* in the water samples ranged from 0.33 to 53.15% with the highest abundance occurring during late summer at the Inlet site (sample RW04). This sample was dominated by the genus *Planktothrix,* which comprised 41.76% of the total microbial community. Meanwhile, the Colony site experienced low cyanobacterial abundance (2.53%), though *Planktothrix* still dominated the cyanobacterial community (32.13%). Cyanobacterial abundance decreased sharply at the Inlet site during autumn (1.97%), though *Planktothrix* and *Microcystis* remained the dominant members of the cyanobacterial community (19.95 and 18.92%, respectively). Meanwhile, cyanobacterial populations grew to 16.82% at the Colony site and the cyanobacterial community became dominated by *Microcystis* (38.52%), while *Planktothrix* populations decreased (7.09%).

In early summer, at the Inlet site, the cyanobacterial community was dominated by microbes from the *Synechococcaceae* family, the most abundant genus being *Vulcanococcus* (38.92%). *Microcystis* populations made up 14.50% of the cyanobacterial community at the Colony site. Winter saw a richness in diazotrophic *Nodularia* species, which comprised 20.52 and 36.25% of the cyanobacterial community at the Inlet and Colony sites, respectively.

Spring saw negligible cyanobacterial populations at both sites; 0.63% at the Inlet and 0.32% at the Colony sites. This season instead showed domination by heterotrophic *Limnohabitans* from the *Proteobacteria* phylum, which comprised 28.68 and 34.58% of the Inlet and Colony populations, respectively, and *Flavobacterium* from the *Bacteroidota* phylum, which comprised 13.29 and 18.17% at the Inlet and Colony sites, respectively.

### MAG dataset

Assembly and binning were undertaken separately for each sample. Two assemblers and three binning algorithms were compared to generate the highest-quality dataset which captured the most bins. Comparison of two assemblers (metaSPAdes [44] and MEGAHIT [45]) found metaSPAdes total contig length was superior to that of MEGAHIT. MetaSPAdes also produced the most contigs ≥50,000 and ≥1,000 bp; however, MEGAHIT produced the most contigs ≥25,000 bp, ≥ 10,000 bp and ≥5,000 bp. MEGAHIT also produced contigs with a greater N50 value in all samples, except RW10 (Data S3). Use of the metaWRAP [46] binning tool resulted in more bins than metabat2 and maxbin2 in every sample and produced more high-quality and marginally less medium-quality bins when supplied with MEGAHIT assemblies. Read assembly using MEGAHIT and contig binning using the metaWRAP tool was therefore pursued in the generation of the MAG dataset.

**Fig. 2.**
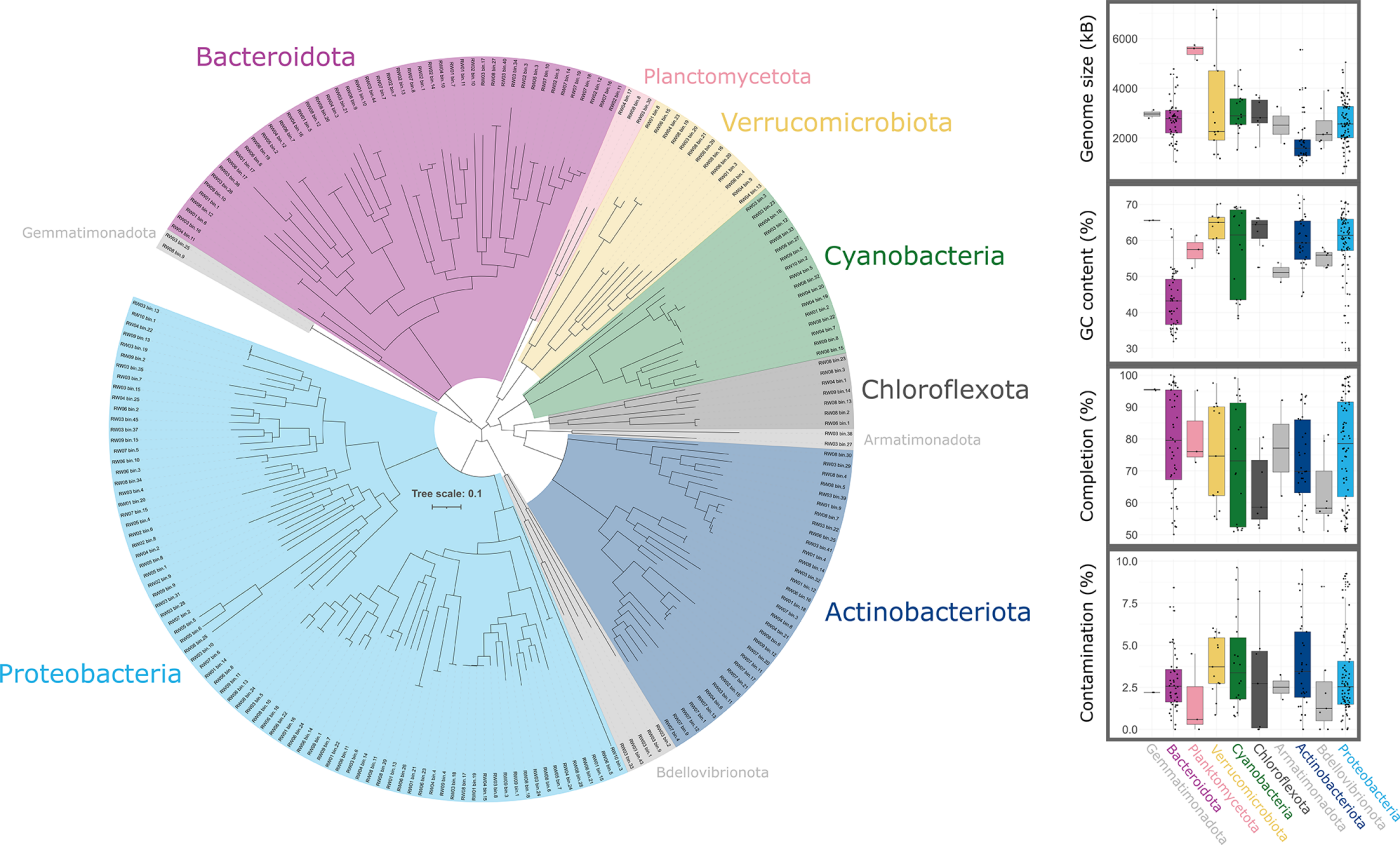
Phylogenetic tree and basic statistics for the MAG dataset. The approximate maximum-likelihood tree was inferred from the concatenated alignment of 74 proteins using FastTree and the GToTree workflow. Branches and clades are coloured and labelled based on the taxonomic annotations estimated by GTDB-Tk. Boxplots showing the distributions of MAG genome size (kB), completeness, contamination and GC content scores are coloured to match the phylogenetic tree. The interactive tree may be accessed here: https://itol.embl.de/tree/1341488223716411687316963.

A total of 216 MAGs were recovered from the 10 shotgun metagenome datasets (Data S5). Of these, 62 were defined as high quality, and 154 defined as medium quality. The total MAG dataset comprised 5.76×10^8^ nucleotides and captured a total of 14 phyla. Most recovered MAGs were classified as *Proteobacteria* (37%), followed by *Bacteroidota* (22%), *Actinobacteriota* (15%) and *Cyanobacteria* (8%). Median completion scores for MAGs in most phyla were ≥70%, except for *Bdellovibrionota* (58%) and *Chloroflexota* (58%), while median contamination scores in all phyla did not exceed 5%. *Bacteroidota* had the lowest median score for GC content (43%), while *Cyanobacteria* had the greatest GC content interquartile range (Fig. 2).

### Taxonomic recovery and GC content effects

To assess taxonomy lost during assembly and binning, bacterial reads were aligned to the MAG datasets. An average of 12.3% of reads aligned to the MAG datasets, ranging from 1% (sample RW05) to 25% (sample RW07). It is acknowledged that this approach captures only the intersection of two independently biassed analyses, that is, read-based taxonomic classification and MAG recovery, and that unclassified reads may also map to the MAG dataset. However, total read-to-MAG mapping was independently assessed via CoverM during relative abundance estimation (Data S5), providing a complementary community-level view that is not limited by classification rate. Most lost information belonged to *Proteobacteria* in almost all samples (Data S6), as this phylum constituted the majority of taxonomic classifications and the highest intra-phyla diversity at the species-level, which complicates high-quality MAG recovery. One sample, RW04, was an exception, where information lost belonged to *Cyanobacteria*.

**Fig. 3.**
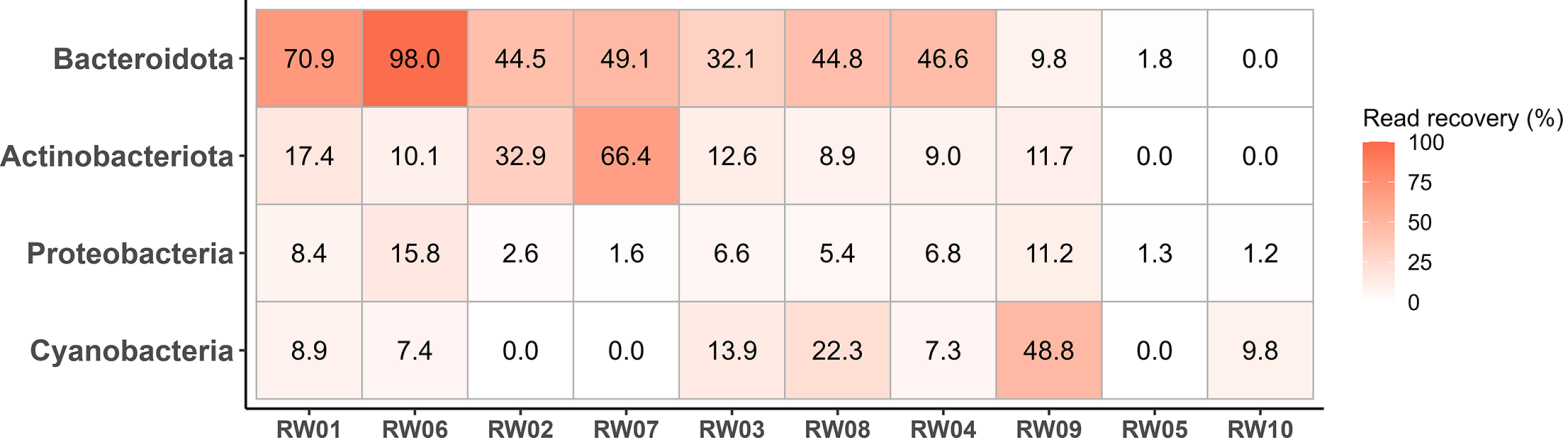
Binning efficiencies within each sample for the four major phyla in this study. Samples are clustered according to sampling date. The numbers within heatmap cells represent binning efficiencies, which here is defined as the percentage of reads for each phylum which could be aligned to the MAG datasets.

Most samples had relatively high binning efficiencies for *Bacteroidota* (Fig. 3), particularly during spring where this phylum was most abundant (samples RW02 and RW07). Binning efficiency here refers to the proportion of reads classified to a given phylum that could be successfully aligned to a MAG from that phylum; a value of 100% would indicate complete recovery of all reads from that taxon into the MAG dataset, while 0% indicates no recovery. Binning efficiencies for *Actinobacteriota* were also notably high in these samples (33 and 66.4% in samples RW02 and RW07, respectively). A higher binning efficiency for cyanobacteria was noted in sample RW09 (49%). Surprisingly, a *Planktothrix* MAG was recovered in this sample (Data S5; RW09_bin.8), though not in the sample where *Planktothrix* was far more abundant (Fig. 1; sample RW04).

**Fig. 4.**
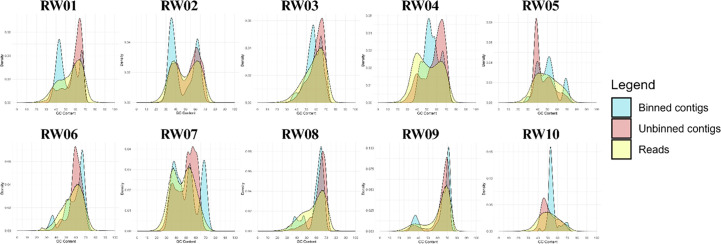
Density plots showing contig and read GC content. The plot includes reads, binned contigs ≥1,000 bp and unbinned contigs ≥1,000 bp collected from all samples. Average GC content is displayed on the x-axis, and plot density is displayed on the y-axis.

The apparent overall higher binning efficiencies of the low GC *Bacteroidota* drove an investigation into the effect of GC content on assembly and binning. GC content was determined for binned and unbinned contigs and reads. Read GC content trended towards 60–65%. Density plots suggested a binning preference for reads with GC content between 55 and 75% (Fig. 4). This trend was consistent in most samples, except samples RW05 and RW10, obtained during summer. It should be noted that samples RW05 and RW10 experienced the lowest classification rates, assembly N50 values and binning efficiencies. The composition of the unclassified read fraction in these samples could not be determined from the database used; however, the convergence of these metrics suggests these samples may have harboured a substantial non-prokaryotic community, and patterns observed within them are interpreted with caution. No obvious trend was observed between GC content and binned contigs, though the density of binned contigs was generally higher than unbinned contigs for GC contents between 35–45%.

**Fig. 5.**
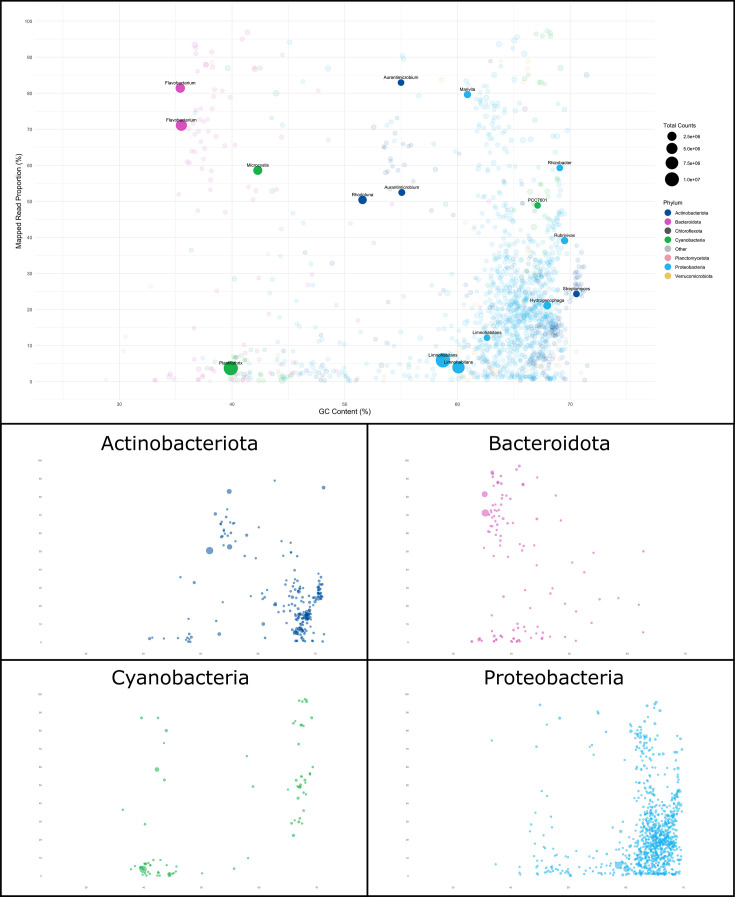
Genus-specific binning efficiencies and GC content. Each bubble in the plot represents reads classified as one genus from one sample. Only genera with at least 10,000 reads are represented in the plot, and genera with at least 1,000,000 reads are highlighted and labelled. The y-axis represents the proportion of aligned vs unaligned reads, meaning higher bubble placement equates to greater MAG recovery. The x-axis represents average read GC content. Bubble size approximates to the sum of mapped and unmapped reads. Additional plots for the four major phyla (*Bacteroidota*, *Actinobacteriota*, *Proteobacteria* and *Cyanobacteria*) were drawn separately to highlight phyla-specific trends.

To investigate a preference during binning for specific taxa, reads with taxonomic annotations were aligned to MAG datasets. The proportion of aligned vs unaligned reads for each genus in each sample was calculated, as well as the average GC content of the reads for each genus (Fig. 5). A slight trend was observed which coincided with lower read GC content and greater MAG recovery. This trend was more pronounced in *Actinobacteriota* and *Bacteroidota*. In particular, *Actinobacteriota* saw poor MAG recovery as GC content trended towards 70%. Conversely, *Cyanobacteria* saw poor MAG recovery as GC content trended towards 40–45%. The most substantial losses in taxa were for the genus *Planktothrix* from the phylum *Cyanobacteria* and the genus *Limnohabitans* from the phylum *Proteobacteria*. Notably, *Limnohabitans* saw substantial abundance (>1,000,000 reads) and poor MAG recovery in three different samples (RW02, RW03 and RW07). Conversely, *Flavobacterium*, the major genus from the *Bacteroidota* phylum, saw high MAG recoveries (>70%) in samples RW02 and RW07.

### Taxonomic recovery through unmatched sample mapping

**Fig. 6.**
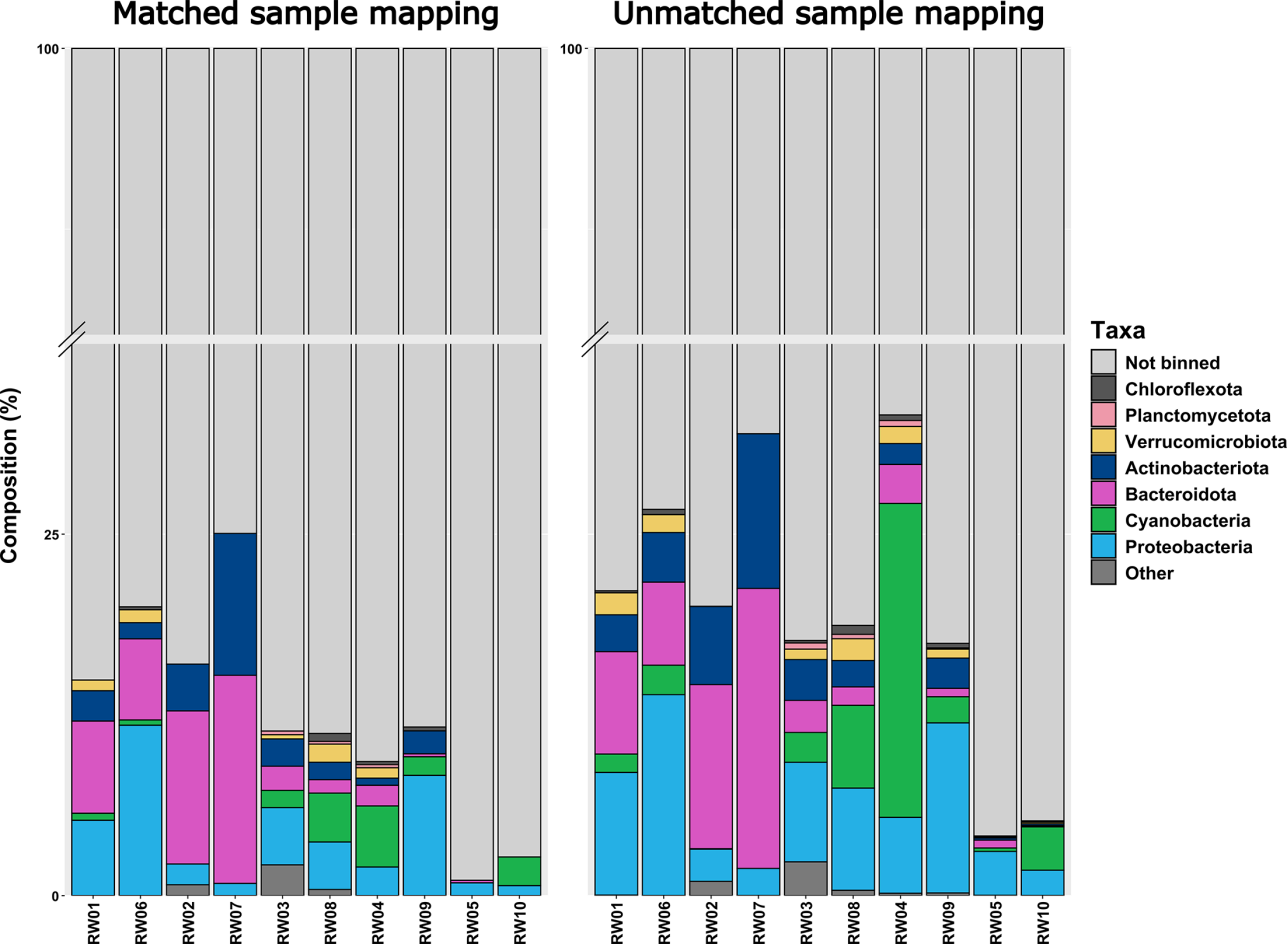
Comparison of matched vs unmatched sample mapping. Composition (%) is on the y-axis and is calculated as the percentage of reads annotated with bacterial classifications. Samples are grouped according to sampling dates. Unmatched sample mapping saw an overall greater recovery in every sample, with the greatest recovery occurring in sample RW04, mostly attributed to *Cyanobacteria*.

To recover taxonomic information lost through assembly and binning, reads from each sample were aligned to the total MAG dataset. On average, an additional 7.3% of classified bacterial reads per sample could be aligned to MAGs recovered from other samples, representing taxonomic information that would otherwise be absent from per-sample MAG datasets (Fig. 6 and Data S7). The greatest recovery occurred in sample RW04 (24%), primarily due to *Cyanobacteria* (17.5%). *Cyanobacteria* also had the greatest recovery summed across samples (25%), followed by *Proteobacteria* (23.9%), *Bacteroidota* (9.6%), *Actinobacteriota* (9.1%) and *Verrucomicrobiota* (3.1%).

*Planktothrix* showed the most substantial recovery due to unmatched sample mapping. The MAG recovered from sample RW09 had a significantly higher abundance in sample RW04 (0.35 and 12.26%, respectively), wherein it also exhibited its highest coverage (~460×). Additionally, 42 other MAGs exhibited greater relative abundance outside of the samples they were originally recovered.

## Discussion

As the recovery of MAGs becomes standard practice for studying microbial ecology, it is important to recognize how it may skew our understanding of microbial diversity. Here, we show that species abundance does not equate to the ease of MAG recovery, as several genera with major representation (namely *Planktothrix* and *Limnohabitans*) were absent from the medium and high-quality MAG datasets. Additionally, we show that species from the *Bacteroidota* phylum were well-represented, as were low-GC species from the *Actinobacteriota*.

A key limitation of this study is the absence of independent, non-sequencing validation of community composition. Metagenomic read-level abundance estimates are subject to sequencing depth limitations and GC-associated bias and should not be interpreted as an absolute measure of true community composition. The biases identified here therefore represent a downstream, assembly- and binning-stage layer of distortion compounding an already imperfect read-level signal, which is a caveat we believe is important to acknowledge given the central role of read-based abundance in the study narrative.

The chosen assembly and binning method yielded 216 medium- and high-quality MAGs, which is an outcome comparable to other studies recovering MAGs from water samples [47,48]. GC content of the recovered cyanobacterial MAGs showed high interquartile range, which is consistent with the literature suggesting fitness within this phylum for a range of ecophysiological conditions [49,50]. Although studies suggest low-GC micro-organisms recovered from metagenomic data experience lower rates of genome completion [15], this was not immediately apparent in this study, and indeed lower GC organisms in the *Bacteroidota* and *Actinobacteriota* phyla experienced greater MAG recovery.

Although <25% of bacterial reads were recovered into MAGs in any sample, this loss is expected and consistent with other studies [51,52]. Samples RW05 and RW10 exhibited the lowest bacterial classification rates, assembly N50 values and binning efficiencies, while also being the only samples whose GC content trended below 50%. Read level taxonomic profiling indicated a high proportion of unclassified reads in these samples. While the precise composition of this fraction could not be determined from the database used, the convergence of low bacterial classification, poor assembly metrics and sub-50% GC content suggests that these samples may have harboured a substantial non-prokaryotic community. Patterns observed within these samples are therefore interpreted with caution and are not considered representative of GC-associated MAG recovery bias in prokaryotes. Where possible, future studies could consider removal of eukaryotic organisms prior to DNA extraction to increase the robustness of MAG recovery efforts [53].

A major finding in this study was that highly abundant micro-organisms in the *Planktothrix* and *Limnohabitans* genera were not recovered into MAGs. This finding contrasts with the notion that MAG recovery efforts are biassed towards more abundant organisms [54,55]. Failure to recover a *Planktothrix* MAG is likely due to a poor assembly, evidenced by a higher density for unassembled reads around the GC content expected for *Planktothrix* (~40%) (Fig. 4; RW04). Additionally, unpublished work attempting to force *Planktothrix* MAG recovery was performed, wherein *Planktothrix*-classified reads from sample RW04 were extracted and assembled. The resulting assembly had a total length of 5.6 Mb; however, only 3.4 Mb was contained in contigs ≥1,000 bp, the minimum size requirement for binning. Given the *Planktothrix* genome size range is between 4.7 and 6.7 Mb [56,57], the assembly was considered inadequate for binning purposes. Nevertheless, a *Planktothrix* MAG was recovered, but it had a completion score <50% and was therefore discarded. The domination of an AT-rich organism such as *Planktothrix* is known to be problematic during sequencing [15] and is likely the reason a *Planktothrix* MAG was not recovered in this sample.

Despite the substantial *Limnohabitans* presence in three samples (Fig. 5), a representative MAG was not recovered. *Limnohabitans* is a diverse genus, and reads were attributed to 30 different *Limnohabitans* species (data not shown). Inspection of metabat2 bins in samples RW02 and RW07 revealed three *Limnohabitans* bins with completeness and contamination scores outside of the acceptable range for MAGs, including one bin with a contamination score of 383% (Data S7). Despite observably different morphologies and phenotypes, *Limnohabitans* species exhibit low genetic diversity [58], making it particularly susceptible to chimeric binning as contigs from divergent strains share sufficient sequence similarity to be incorrectly co-binned.

Notably, *Limnohabitans* species can indicate a collapsed cyanobacterial or algal bloom, as the genus thrives on the exudates of phototrophic microbes [59]. These ecological disturbances, as well as others such as eutrophication or sediment mixing, create niche conditions which drive strain-level genetic diversity [60,62]. Results collected here suggest microbes with pertinent functional roles may be lost due to MAG heterogeneity in similar studies, especially those with higher GC content. GC content is known to bias sequencing efforts, as GC-rich and GC-poor sequences respond poorly to library preparation and amplification protocols required by next-generation sequencing platforms [10,63].

A trend was observed between lower GC content and higher MAG recovery in *Bacteroidota* and *Actinobacteriota*. Notably, the genus *Flavobacterium* in the phylum *Bacteroidota* demonstrated a high recovery rate in two samples (RW02; 83% and RW07; 73%). The MAGs recovered from this genus exhibited low GC ranging from 32 to 37%, while also exhibiting small genome sizes, averaging 2.1 Mb. Similarly, the efficiently recovered *Aurantimicrobium* and *Rhodulana* genera from the phylum *Actinobacteriota* exhibited a combined average GC content of 54% and genome size of 1.2 Mb, compared to 62% and 2.2 Mb in other *Actinobacteriota* genera, which were poorly recovered. These findings suggest that while GC content does not directly impact unbiased binning algorithms, it does influence genome size, facilitating the recovery of MAGs with lower GC content. This is consistent with recent findings linking lower GC content to smaller chromosome sizes in bacteria [64]. Collectively, these results indicate that MAG recovery efforts may be biassed towards smaller, AT-rich genomes, which in this study resulted in the over-representation of species in the *Bacteroidota* and *Actinobacteriota*. Future studies could explore the influence of GC content and genome size across more diverse real-world samples to understand the broader implications of this bias during MAG recovery.

We demonstrate that taxa can be recovered by mapping unmatched samples against the entire MAG dataset. By measuring the relative abundance of MAGs in this manner, we identified *Planktothrix* as the causative agent of a late summer bloom at the Inlet site. This identification is consistent with independent 16S rRNA amplicon sequencing of the same environment and overlapping sampling period, which reported *Planktothrix* as a dominant taxon during late summer [18], providing independent biological support for the read-level abundance profiles central to this study’s narrative. While co-assembly is another method to resolve genomes present across multiple samples, it requires substantial RAM [9] which may not be feasible in all circumstances. Additionally, co-assemblies have the potential to produce more heterogeneous or erroneous MAGs by binning similar genomic fragments from distinct organisms [65]. The approach demonstrated here will enable other researchers facing similar challenges to resolve more taxa, despite restricted computational infrastructure.

The recovery of MAGs from metagenomic sequencing data has become standard practice in microbial ecology, significantly enhancing our understanding of microbial diversity. In this study, we demonstrate that species abundance does not necessarily correlate with ease of MAG recovery. Our findings show the efficient recovery of smaller, low-GC microbes, particularly over-representing species from the *Bacteroidota* and *Actinobacteriota*. Such identifications are crucial for addressing questions related to functions such as substrate use and microbial interactions. While MAG recovery is a powerful and essential technique, we hope this study encourages microbial ecologists to adopt similar investigations during MAG recovery efforts. Such practices will ultimately provide insights into how MAG recovery may skew the representation of specific taxa on a global scale. It should be noted that the mean number of reads obtained in this study (42.9 M) was lower than that obtained in similar studies comparing different binning approaches. Increased sequencing depth undoubtedly improves assembly and MAG recovery efforts and should be considered if the pipeline in this study is employed.

In conclusion, this study demonstrates that species abundance does not equate to ease of MAG recovery, with highly abundant genera such as *Planktothrix* and *Limnohabitans* absent from the MAG dataset despite their dominance in read-based community profiles. Recovery bias was observed towards smaller, lower-GC genomes, particularly those of *Bacteroidota* and *Actinobacteriota*, and is likely driven by a combination of GC-associated sequencing bias, genome size and strain-level heterogeneity acting as compounding determinants of assembly and binning success. We further demonstrate that unmatched sample mapping can partially recover taxonomic information lost during per-sample MAG recover*y*. The pipeline is deployable on modest workstation-based infrastructure, making it accessible to research groups without access to high-performance computing facilities. We hope this study and the accompanying pipeline encourage microbial ecologists to critically evaluate MAG recovery outputs and to recognize that the organisms most ecologically significant in a given system may not always be those most amenable to genome-resolved analysis.

## Supplementary material

10.1099/mgen.0.001736 Supplementary Material 1.

10.1099/mgen.0.001736 Supplementary Material 2.

## Abbreviations

MAG: metagenome-assembled genome
