# Supplementary material for: Taxonomy bias in metagenome-assembled genome recovery
Source: Microb Genom. 2026 Jun 8;12(6):001736. doi: 10.1099/mgen.0.001736 (PMC13245658; doi:10.1099/mgen.0.001736)
Supplement: Supplementary Material 1. [file mgen-12-01736-s001.pdf]

Description of Additional Supplementary Files

Supplementary Data 1

Description: Metagenomic read sequence counts and % GC.

Supplementary Data 2

Description: Metagenomic read classification rates.

Supplementary Data 3

Description: Results comparing assembly success. MS = metaSPAdes. MH = MEGAHIT.

Supplementary Data 4

Description: Results comparing binning success. The metaSPAdes assemblies were used to determine the binning method which yielded the greatest number of medium and high-quality bins. The metaSPAdes and MEGAHIT assemblies were then both compared against the best binning method. MS = metaSPAdes. MH = MEGAHIT.

Supplementary Data 5

Description: MAG descriptions and information. The data reports MAG completion, contamination, GC content, N50 values, size in bp, taxonomic classification and abundance in each sample. Green highlighting represents high-quality MAGs, and yellow medium-quality. MAG abundance was calculated for individual samples as the number of mapped reads divided by total reads.

Supplementary Data 6

Description: Taxonomic information lost due to binning. Reads were mapped to MAGs, and mapped reads compared with those classified with taxonomy to determine taxonomic loss at the phyla level. Loss due to binning is reported with respect to the proportion of reads already classified with taxonomic information, i.e. reads without taxonomic information are ignored.

Supplementary Data 7

Description: Taxonomic information recovered due to unmatched sample mapping. In unmatched sample mapping, abundance within each sample is calculated by mapping sample reads against the whole MAG dataset. Taxonomic recovery is calculated by comparing the summed abundance for each phylum in each sample following unmatched sample mapping with that for matched sample mapping.

Supplementary Data 8

Description: Three *Limnohabitans* bins were recovered, however were of too low quality to be of use in this study.
